# An Abiotic Glass-Bead Collector Exhibiting Active Transport

**DOI:** 10.1038/srep14348

**Published:** 2015-09-21

**Authors:** Youhei Goto, Masato Kanda, Daigo Yamamoto, Akihisa Shioi

**Affiliations:** 1 Department of Chemical Engineering and Materials Science, Doshisha University, Tatara Miyako-dani 3-1, Kyotanabe, Kyoto 611-0321, Japan

## Abstract

Animals relocate objects as needed by active motion. Active transport is ubiquitous in living organisms but has been difficult to realize in abiotic systems. Here we show that a self-propelled droplet can gather scattered beads toward one place on a floor and sweep it clean. This is a biomimetic active transport with loadings and unloadings, because the transport was performed by a carrier and the motion of the carrier was maintained by the energy of the chemical reaction. The oil droplet produced fluctuation of the local number density of the beads on the floor, followed by its autocatalytic growth. This mechanism may inspire the technologies based on active transport wherein chemical and physical substances migrate as in living organisms.

Animals relocate objects as needed by active motion; even in human bodies, liposomes make use of active transport to move chemicals with molecular motors in the cytoskeleton^1^,^2^. Some of man-made active transport systems already proposed use a ratchet mechanism. A saw-tooth-shaped potential and/or its periodic variation rectifies random motion or alternates the current of particles so as to develop irreversible (one-way) flow^3^,^4^,^5^,^6^. The ratchet mechanism is one of the principles that can be used to obtain one-way flow from random motions and may be associated with some vital mass-transports in living organisms^7^,^8^,^9^. Catalytic particles driven by chemical reactions can migrate irrespective of the gradient of chemical potential of the particles^10^,^11^,^12^,^13^,^14^,^15^. Non-equilibrium systems such as a reaction diffusion system enable active transport of the object such as small floating object on BZ solution^16^ and solid soap with Marangoni effect^17^. For a transport system with a semblance of life, however, both loading of transported matter into the carrier and unloading at the target place are essential in addition to the active motion of the carrier itself. These loadings and unloadings are not difficult in our everyday life; this is seen even on the microscopic scale with vesicle transport in a living cell^18^,^19^. However, abiotic transport systems with loading and unloading have not been reported to date except for the use of robots, because, in previous studies^3^,^4^,^5^,^10^,^11^,^12^,^13^,^14^,^15^,^16^,^17^, the carriers were not used.

In this article, we show that a self-propelled droplet can gather scattered beads toward one place on a floor and sweep it clean. Neither the entropy of the bead distribution nor the interaction between beads and the floor surface govern the dynamics. Rather, this surprising bead-transport phenomenon is produced by small, non-thermal fluctuations in the local number density of beads and its autocatalytic growth, both of which are generated by nonlinear droplet dynamics in a strongly non-equilibrium state. This may provide an avenue for designing active transport systems in the laboratory. Technologies based on active transport may produce highly sophisticated micro/nano systems wherein chemical and physical substances migrate as in living organisms.

## Results

We attempted to produce an active transport system with loading and unloading using a simple oil/water system. An oil droplet containing iodine and iodide anion(s) spontaneously moves on a glass surface in water containing a cationic surfactant, trimethyloctadecylammonium chloride (C_18_TACl)^20^,^21^,^22^.

**Figure 1:**
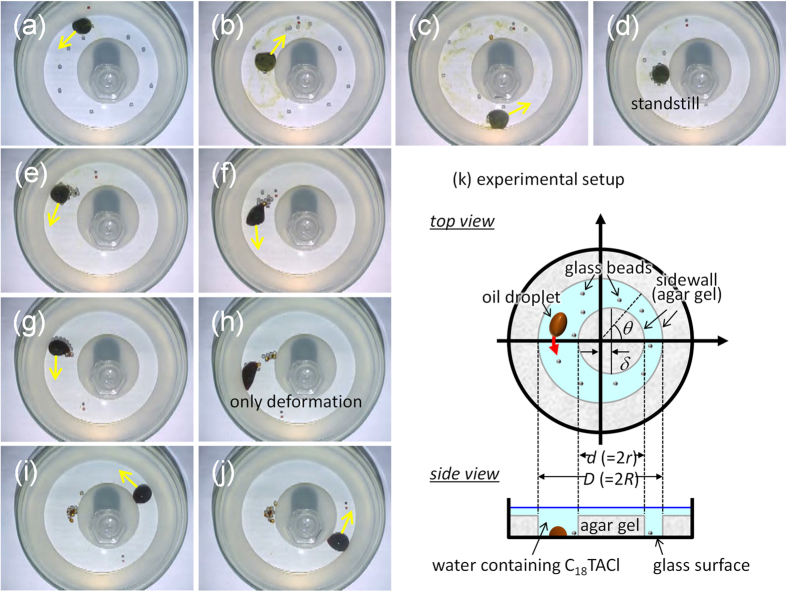
Experimental setup and results for bead transport. In (**a–j**), snapshots are shown every 30 s. The yellow arrow indicates the direction of droplet motion. The diameter of the Petri dish was 9 cm. (**k**) Illustration of the top and side views of the experimental setup. The direction of droplet motion sometimes changes in a sporadic manner.

When the glass beads and the oil droplet were placed on the glass surface, the droplet propelled itself and subsumed the beads. This uptake was a stochastic process. Once a bead was taken up, the droplet retained the bead for some time. After a certain period, the droplet released the bead in a sporadic manner. The uptake and release of a glass bead repeated many times (see Supplementary Video 1). We produced an annular course (a ring) as shown in Fig. 1 by applying a gel to define inner and outer boundaries of the course. When an oil droplet was placed in the ring, it moved clockwise or counterclockwise. When numerous glass beads had been put in the ring, they were carried via the uptake and release by the moving droplet.

**Figure 2:**
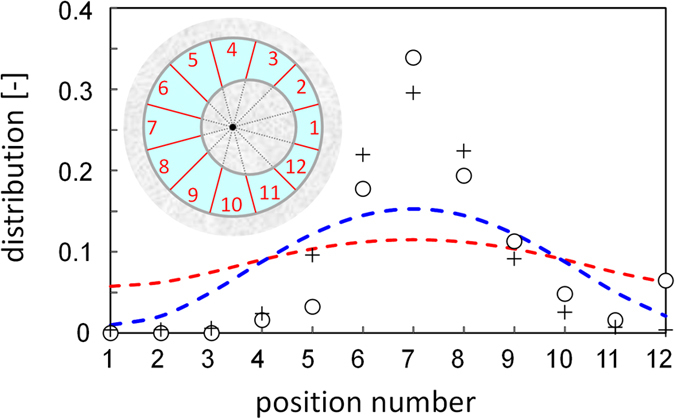
The distribution of the number of the beads at the final state. The ring course is divided into twelve sections shown inset. The abscissa is the position number. The circle is the number of beads at corresponding positions divided by the number of beads on the ring course, which excludes the number of beads contained in the oil droplet. The plus symbol denotes the result of the calculation. See Eqs. (s3.1) and (s3.2) and Fig. S3. The dashed curves are calculated by the maximum entropy principle, eq.s1.1 (red) and eq.s1.2 (blue). (see Supplementary Note 1). The droplet diameter used for eq.s1.2 was 0.9 cm.

When the inner and the outer sidewalls of the ring were concentric, the droplet gathered the beads at random sites (see Supplementary Video 2). However, when the ring was not concentric, the droplet gathered all beads at the widest place in the course (see Supplementary Video 3). Figure 1a–j show snapshots of the beads’ transport for *w*_*r*_ = 2.0, where *w*_*r*_ is the ratio between the widest and narrowest widths in the ring. We repeated the experiments seven times so that a number distribution of glass beads was obtained. The final distribution at 300 s when the droplet motion stopped, is shown in Fig. 2 (open circle). The droplet gathered all beads at the widest places (positions 6, 7, and 8 inset) with extremely high probability. The distribution corresponding to the uniform surface density is also shown (dashed curves of Fig. 2: Red and blue curves correspond to the two types of the evaluation methods of the surface area. See Supplementary Note 1). This is the maximum-entropy distribution. The peak of *w*_*r*_ = 2.0 was much greater than the distribution maximum that the maximum-entropy principle predicts.

If beads are gathered at a narrow site in the course, the moving droplet collides with the beads. On the other hand, a droplet can pass through without collisions when the beads are in wider places. This may be associated with the distribution maximum at *w*_*r*_ = 2.0. However, this geometrical effect is not essential to reaching the final distribution. After the beads had been gathered at the widest place, we moved the inner gel region in such a way that the place occupied with the beads became the narrowest in the course (the plus/minus of *δ* value of Fig. 1k was reversed). Despite contact between the beads and a droplet, almost all of the beads were present in the narrower places (Supplementary Video 4). If the droplet continued to move, the beads might be gathered to the widest place. In this experiment, however, the aggregation that was already completed at the initial state appeared to retard the active transport process. This suggests that aggregate growth process is required for the active transport within the lifetime of a moving droplet and that the active transport shown in Fig.1 could not be explained only by the simple geometrical effect. These considerations demonstrate that the formation of the distribution peak at *w*_*r*_ = 2.0 is governed not by the stability of final positions but by the dynamics required to reach the state: neither entropy of the bead distribution nor interaction between the beads and the glass surface can explain the final distribution at *w*_*r*_ = 2.0.

**Figure 3:**
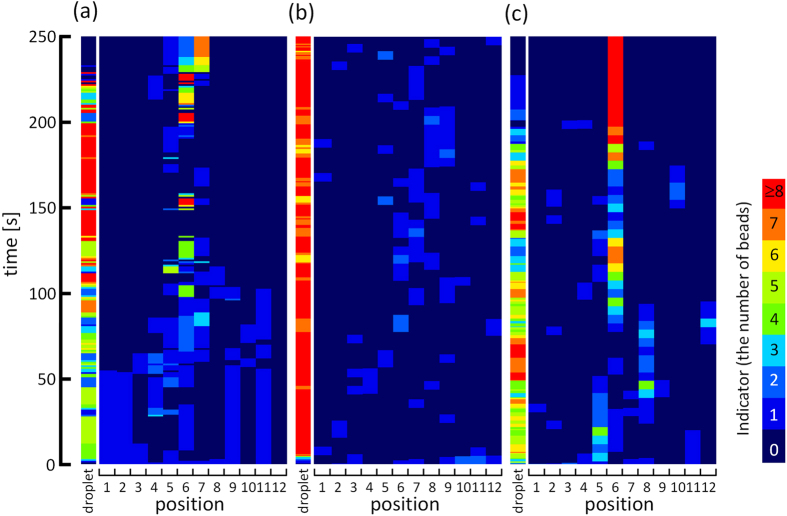
Spatiotemporal plot of the number of the beads. (**a**) Experimental results are shown for a total bead number of ten. The abscissa is the position number shown inset in Fig. 2. The color indicates the number of beads at corresponding positions and times. The slender column at the left of the spatiotemporal plot shows the time evolution of the number of beads contained in the oil droplet. The ordinate is the elapsed time. (**b,c**) show results of the calculations. (**b**) shows the result with the width-dependent *P*_in_ and *P*_out_. (**c**) shows the result with *P*_in_(*n*) and *P*_out_(*n*).

Figure 3a shows the time evolution of the number of beads at each location in the ring. The ring is divided into twelve sections shown in the inset of Fig. 2. The number of the beads near the widest place (position 6) fluctuated significantly compared with that of the other positions. At approximately 200 s, the number of beads near the widest place begins to increase to reach the final distribution. The larger fluctuation near the widest place and its growth are highly reproducible at *w*_*r*_ = 2.0 (see Supplementary Fig. S1).

## Discussion

We performed a simple calculation for this bead transport behavior. Consider a point (droplet) moving on a circle. This circle was divided based on its azimuth into twelve sections by Δ*θ* = 2π/12, and ten beads were distributed randomly in the twelve sections. The droplet moved from one section to the next. When the droplet entered a new section occupied by a bead, the droplet took it up with a probability *P*_in_. When two or more beads were in the section, each bead was taken up independently. On the other hand, when the droplet contained a bead, the bead was released at a probability *P*_out_. When the droplet had two or more beads, each bead was released independently. The calculation with a constant *P*_in_ and a constant *P*_out_, as expected, provided fluctuations of the number of beads everywhere in the ring (Supplementary Fig. S2a).

**Figure 4:**
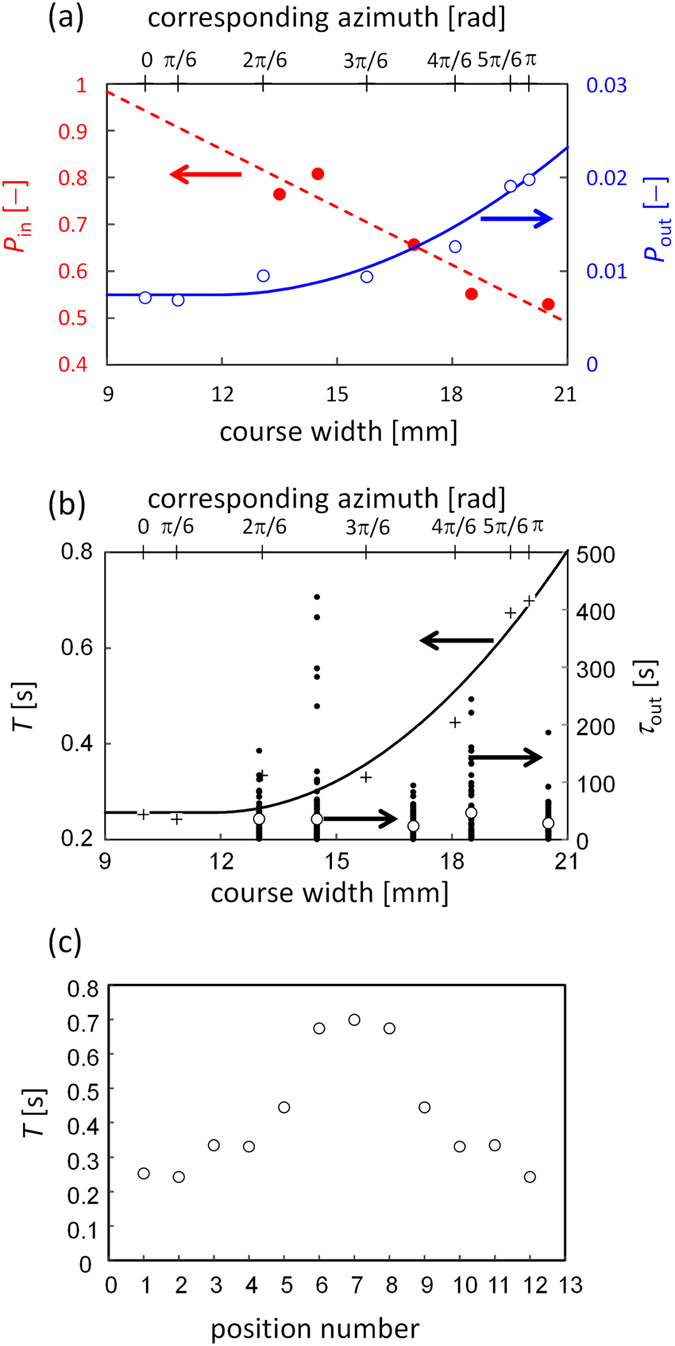
The probability of uptake and release. (**a**) Depicts *P*_in_ (red key) and *P*_out_ (blue key) as functions of the course width. In (**b**) *T* (plus) and *τ*_out_ (black dot) are shown. The open circle represents the arithmetic mean of *τ*_out_, which is approximately 34.2 s irrespective of the width. For the course shown in Fig. 1k, the width varies with the azimuth. The azimuth is shown in the upper abscissa at the position corresponding to its width. The solid curves are the correlations. (Supplementary Note 3) (**c**) *T* is shown against the position number in the inset of Fig. 2. The summation of all *T* values is equal to the average lap time for circular motion of the droplet.

Figure 3a demonstrates that the fluctuation is larger at the wider positions. This suggests that *P*_in_ and *P*_out_ depend on the course width. Thus, experiments to measure of these probabilities were carried out. We placed an oil droplet in the concentric ring and changed the course width. Five glass beads were distributed randomly on the course. We counted the events where the droplet passed through a section occupied by the bead(s). The number of events is *N*_total_. In some cases, the droplet took up the bead(s). The number of uptake events was denoted as *N*_uptake_. Figure 4a shows the *P*_in_ ( = *N*_uptake_/*N*_total_) as a function of the course width. *P*_in_ increases with a decrease in the width because the collision probability between the droplet and the bead(s) is larger in a narrower course.

*P*_out_ at a section with the residence time *T* is given by 1−exp(−*T*/*τ*_out_) (see Supplementary Note 2). *P*_out_ is the release probability at each section and not defined by a unit time. For an evaluation of *τ*_out_, we used the same experimental setup with the concentric ring. After the droplet had taken up the bead, we measured the period for the droplet to retain the bead without releasing it (*τ*_out_). A longer *τ*_out_ value leads to smaller *P*_out_. Figure 4b shows these retention values against the course width. The data are scattered, and their average does not depend on course width. *P*_out_ also depends on the residence time *T* of the moving droplet in each section: in wider positions, a droplet can move along the radial direction in addition to the circumferential one. This degree of freedom in the droplet’s motion increased residence time in the wider sections. Using the course with *w*_*r*_ = 2.0, the residence time when the droplet resided transiently in each section was measured. The average residence time *T* is shown in Fig. 4c with respect to the position number and in Fig. 4b with respect to the course width, indicating that the residence time is longer at wider positions. The resultant *P*_out_ was shown in Fig. 4a. *P*_out_ increases with an increase in the width. This characteristic results from the width-dependency of *T*, while *τ*_out_ is irrespective of the width.

Calculations were performed with the correlation curves for *P*_in_ and *P*_out_ shown in Fig. 4a (Supplementary Note 3). The result is shown in Fig. 3b, demonstrating that the fluctuation in the number of beads appears to be concentrated near the widest place (other examples of the results are shown in Fig. S2b). However, growth of the fluctuation never occurred. This is because the uptake and the release in the calculation are reversible.

When two or more beads were contained in a droplet, they tended to form an aggregate owing to the presence of oil. Once the aggregate had been released, the droplet rarely took up the aggregated beads (Supplementary Video 5). This means that *P*_in_ should be a decreasing function of the aggregate size *n*. Thus, the probability should be denoted as *P*_in_(*n*), where *P*_in_(1) is equal to the *P*_in_ shown in Fig. 4a. Moreover, the bead(s) being carried by a droplet tended to be trapped by the aggregate in the ring (Supplementary Video 6). After mechanical trapping by the large aggregate, the bead(s) adhered to the aggregate owing to oil staining. This indicates that *P*_out_ should be an increasing function of the aggregate size. Here, the probability should be denoted as *P*_out_(*n*), where *P*_out_(0) is equal to the *P*_out_ shown in Fig. 4a. In the present calculation, a linear approximation that satisfies the above conditions was used for simplicity (see Supplementary Note 4).

Figure 3c shows the result calculated with *P*_in_(*n*) and *P*_out_(*n*); other examples are shown in Fig. S2c. The calculation was performed over 50 laps that approximately corresponds to 250 s of the experiments, because the average period required for the droplet to complete one lap was approximately 5 s. The fluctuation and its growth are similar to the experimental results. The average distribution of the beads was calculated from 10 000 simulations and is shown in Fig. 2. The simulation reproduces the experimental results well. The functional form of the *n*-dependency in *P*_in_(*n*) and *P*_out_(*n*) does affect the quantitative aspect of the results. However, most important is that both *P*_in_(*n*) and *P*_out_(*n*) provide autocatalytic growth of the fluctuation of the number of beads: the growth of the cluster is accelerated with an increase in cluster size.We found an abiotic active transport system with loading and unloading. The glass beads are transported to the widest place by a self-moving droplet that carries the beads. This phenomenon can be explained by autocatalytic accumulation seeded by random release. This scenario may be developed to design the transport systems with a semblance of life and their applications to future technologies, such as active transport in microfluidic devices.

## Methods

### Materials

Trimethyloctadecylammoniumchloride (purity >98.0%) was purchased from Tokyo Chemical Industry Co., Ltd. Nitrobenzene (purity >98.0%), potassium iodide (purity >99.9%), iodine (purity >99.8%), and agar of reagent grade were provided by Wako Chemicals Inc. All chemicals were used without further purification. Glass beads of 2 mm were provided by AS ONE Corporation. All glass beads and Petri dishes were treated by vacuum plasma so as to remove surface contamination (FEMTO Science CUTE-MP(MP/R)). After treatment, the glass surfaces were wetted with KOH aqueous solution (1 M) and rinsed with deionized water.

### Procedures

An aqueous solution containing 4% agar was poured into a Petri dish 9 cm in diameter. The initial temperature of the solution was approximately 80 °C and it was allowed to cool to room temperature. Special care was taken not to introduce bubbles to the gel. After gelation, a cylindrical gel mass of diameter *D* (<9 cm) was cut out using a cookie cutter. Next, a ring-shaped gel was obtained, with inner and outer diameter equal to *D* (cm) and 9 cm, respectively. A cylindrical gel of diameter *D* was also obtained. From the cylindrical gel, another cylindrical gel with diameter *d* (<*D*) was cut out by a cookie cutter. Next, two pieces of gel, the ring-shaped gel and the cylindrical gel with the diameter *d*, were obtained. These gels were soaked in an aqueous solution of C_18_TAC (3 mM). The aqueous solution was refrigerated for 24 hrs. After treatment, the gels were removed from the refrigerator and washed in deionized water. These gels were put in a Petri dish of 9-cm diameter so as to form the desired layout. The central gel shown in Fig. 1k was weighted to prevent its movement due to droplet motion. In the typical experiment, the inner (*d*) and the outer (*D*) diameters were 3.4 cm and 6.4 cm, respectively. Thus, the widest width *L*_max_ was related to *w*_*r*_ by *L*_max_ = {(*D*–*d*) *w*_*r*_} / (1 + *w*_*r*_). The *L*_max_ of *w*_*r*_ = 2.0 was 2.0 cm; this was approximately two times larger than the average diameter of an oil droplet (approximately 0.8–1 cm). The aqueous solution containing trimethyloctadecylammonium chloride (3 mM C_18_TAC) was poured into the ring-shaped course so that the depth of the C_18_TAC-containing aqueous solution was around 20 mm: that is, a large enough depth compared to the droplet’s diameter. The glass beads of 2-mm diameter were placed randomly in the ring course filled with the aqueous solution. The number of beads was usually ten. This was nearly the maximum number of the glass beads, because too many beads restricted the free motion of the oil droplet. After setting, a nitrobenzene droplet that contained 50 mM I_2_ and saturated KI was placed in the ring. The droplet volume was 400 μL, and its diameter was approximately 8–10 mm. The experimental result was recorded by a CCD camera (Keyence Corporation VW-6000/5000), and the movie was analyzed by the software MovieRuler (Photron limited).

## Additional Information

**How to cite this article**: Goto, Y. *et al.* An Abiotic Glass-Bead Collector Exhibiting Active Transport. *Sci. Rep.* **5**, 14348; doi: 10.1038/srep14348 (2015).

## Supplementary Material

Supplementary Information

Supplementary Video 1

Supplementary Video 2

Supplementary Video 3

Supplementary Video 4

Supplementary Video 5

Supplementary Video 6
