# Supplementary material for: An Abiotic Glass-Bead Collector Exhibiting Active Transport
Source: Sci Rep. 2015 Sep 21;5:14348. doi: 10.1038/srep14348 (PMC4585695; doi:10.1038/srep14348)
Supplement: Supplementary Information [file srep14348-s1.pdf]

Abiotic Glass-Beads Collector Exhibiting Active Transport

Youhei Goto, Masato Kanda, Daigo Yamamoto, and Akihisa Shioi*
Department of Chemical Engineering and Materials Science, Doshisha University
Tatara Miyako-dani 3-1, Kyotanabe, Kyoto 611-0321, Japan

Supplementary Video 1

Moving oil droplet taking up and releasing a glass bead. The concentrations of I_2 and C_{18}TAC are 20 mM and 1.5 mM, respectively. Ten times faster than the real speed.

Supplementary Video 2

Four examples of the beads transport at $w_r = 1$. The concentrations of I_2 and C_{18}TAC are 50 mM and 3.0 mM, respectively. Ten times faster than the real speed.

Supplementary Video 3

Four examples of the beads transport at $w_r = 2$. The concentrations of I_2 and C_{18}TAC are 50 mM and 3.0 mM, respectively. Ten times faster than the real speed.

Supplementary Video 4

Experimental result of the beads transport with the variation in the course width. At the initial state, the glass beads are gathered at the widest place, but the place becomes narrowest by moving the central gel. The aggregate of the beads do not move to the new widest place. The concentrations of I_2 and C_{18}TAC are 50 mM and 3.0 mM, respectively. Twenty times faster than the real speed.

Supplementary Video 5

The oil droplet rarely carries the aggregate of glass beads despite the contacts. The concentrations of I_2 and C_{18}TAC are 50 mM and 3.0 mM, respectively. This is the real time video.

Supplementary Video 6

The oil droplet gathers all the glass beads toward an aggregate, despite that the aggregate is not placed at the widest position. The concentrations of I_2 and C_{18}TAC are 50 mM and 3.0 mM, respectively. Five times faster than the real speed.

Supplementary Note 1

The maximum entropy principle predicts that a surface density of the glass beads should become uniform independently of the position. Then, the distribution is proportional to d$S(\theta)$/dθ, where d$S(\theta)$ is the area of a section between θ and θ + dθ in the ring. The function d$S(\theta)$/dθ is given by

$$\frac{dS(\theta)}{d\theta} = \frac{R^2}{2}\left[1-\left\{\frac{\delta}{R}\cos\theta+\sqrt{\left(\frac{r}{R}\right)^2-\left(\frac{\delta}{R}\right)^2\sin^2\theta}\right\}^2\right] \quad \text{(s1.1)}$$

where $R = D/2$ and $r = d/2$.

We normalized the right hand side of eq.s1.1 such that the integration from 0 to 2π becomes unity. The experimental distributions are presented by each $2\pi/12$, and hence we multiplied the normalized dS(θ)/dθ by $\pi/6$. A red dashed curve of Fig. 2 is a result of this calculation.

We may consider that $S(\theta)$ should be calculated with the rest area that is not occupied by the moving droplet. The rest area between θ and θ + dθ is given

$$\frac{dS(\theta)}{d\theta} = \frac{R^2}{2}\left[1-\left\{\frac{\delta}{R}\cos\theta+\sqrt{\left(\frac{r+D_p}{R}\right)^2-\left(\frac{\delta}{R}\right)^2\sin^2\theta}\right\}^2\right] \quad \text{(s1.2)}$$

Here, D_p is the droplet diameter.

Supplementary Note 2

The probability for releasing a bead is *pdt* that is proportional to a time interval *dt*. Here, *p* denotes a constant. Then, the probability for a droplet to keep a bead is 1– *pdt*.

Consider a finite time interval T. Then, the probability for a droplet to keep a bead during this time interval is given by $(1- pdt)^{T/dt}$. Thus, the probability for releasing a bead within the interval T, that is P_{out}, is given by $1-(1- pdt)^{T/dt}$. Taking a limit of $dt \to 0$ yields

$P_{out} = 1- \exp(- pT)$ (s2.1)

The mean period for a droplet to keep a bead (τ_{out}) is calculated to be

$$\tau_{out} = \int_0^\infty (1 - P_{out}) dT = p^{-1} \qquad \text{(s2.2)}$$

Thus, P_{out} is given by

$P_{out} = 1- \exp(-T/\tau_{out})$ (s2.3)

Supplementary Note 3

We use the correlation curves of P_{in} and P_{out} for calculations. The P_{out} is calculated from the correlation curves of T and τ_{out}. The experimental data of P_{in} and τ_{out} were obtained in the width range between 13.0 and 20.5 mm. A linear dependency of P_{in} on the course width and an approximately constant τ_{out} (34.2 s) were obtained. These dependencies were used for all the width values required for the calculation. The residence time T was evaluated with a ring course shown in Fig.1. In this experiment, T value was obtained for the course width between 10.0 and 20.0 mm. This result was correlated by an empirical equation. Resultantly, all the equations used for the calculation are

$P_{in} = -0.0411x+1.3529$

$P_{out} = 1 - \exp[-T/\tau_{out} (=34.2\ \text{s})]$

$T = 0.0065x^2-0.1537x+1.1652$ ($x > 11.82038$)

$=0.256597$ ($x < 11.82038$),

where x denotes the course-width. The calculated curves are shown in Fig.4.

Supplementary Note 4

We assume the linear function by

$P_{in}(n) = P_{in}(n-n_{cr})/(1-n_{cr})$ at $n \le n_{cr}$, and 0 at $n > n_{cr}$ (s3.1).

$P_{out}(n)=(1-P_{out})(n-m_{cr})/m_{cr}+1$ at $n < m_{cr}$, and 1 at $n > m_{cr}$ (s3.2).

They are shown in Fig.S3. Here, n_{cr} and m_{cr} are adjustable parameters.

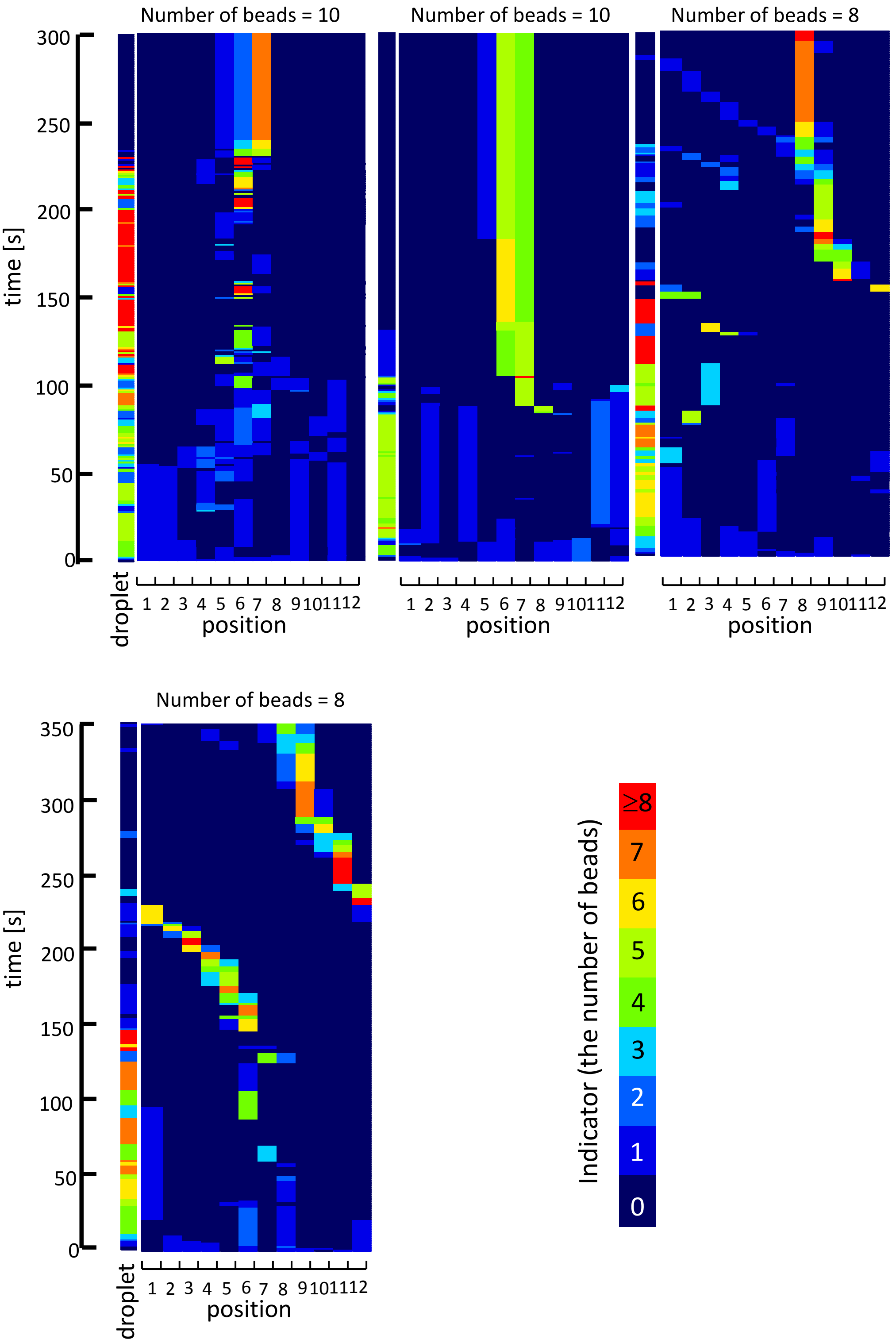

Figure S1

Spatiotemporal plot of the number of the beads at the divisions shown in the inset of Fig.2. The experimental conditions are the same as that for the Fig.3a. Because the beads transport is a stochastic process, the examples besides Fig.3a are shown. Details for this presentation are explained in the caption of Fig.3.

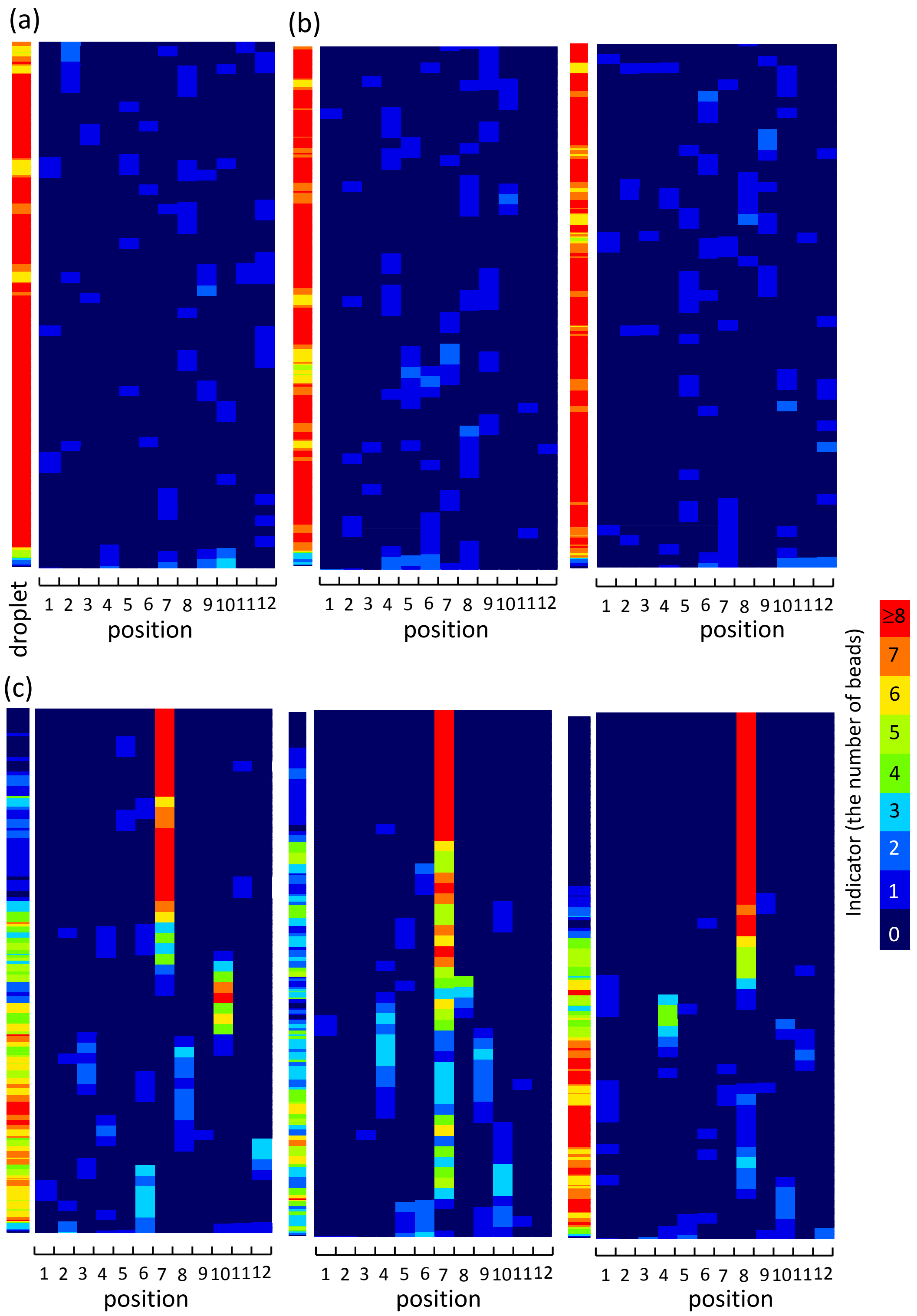

Figure S2

Spatiotemporal plot of the calculated distribution of *beads*. (a) P_{in} = 0.6 and P_{out} = 0.01. (b) The results of the calculation with the width-dependent P_{in} and P_{out}. (c) The results the calculation with $P_{in}(n)$ and $P_{out}(n)$. Details for this presentation are explained in the caption of Fig.3.

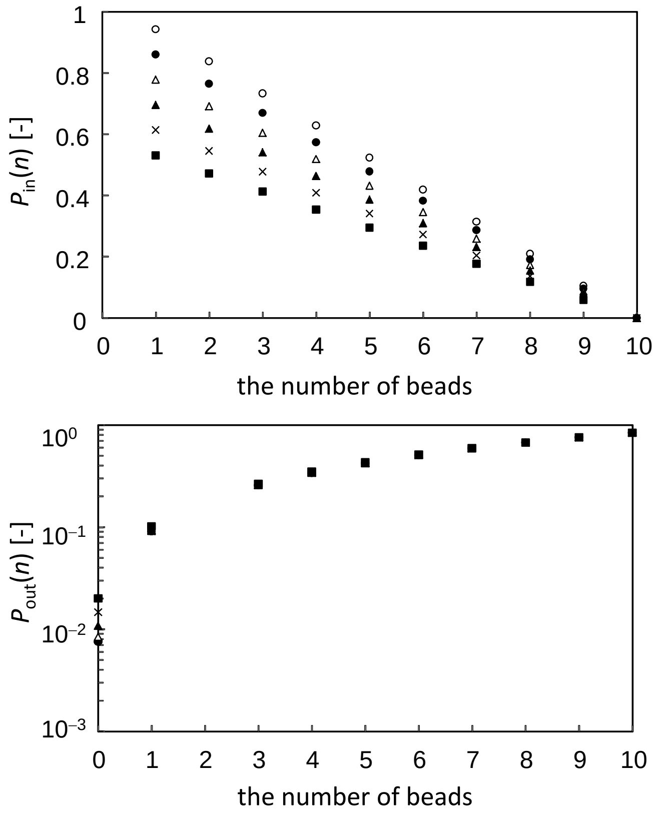

Figure S3

$P_{in}(n)$ and $P_{out}(n)$ used for the calculation of Fig.3c. The abscissa is the value of n. Keys represent the course width: They are 10 mm (open circle), 12 mm (closed circle),14 mm (open triangle), 16 mm (closed triangle),18 mm (cross), and 20 mm (square). They are nearly overlapped in $P_{out}(n)$ except for n=0. The equations are shown in Supplementary Note 4. The parameters of n_{cr}=10 and m_{cr}=12 are used.
